# Dental Caries Prediction Based on a Survey of the Oral Health Epidemiology among the Geriatric Residents of Liaoning, China

**DOI:** 10.1155/2020/5348730

**Published:** 2020-12-07

**Authors:** Lu Liu, Wei Wu, Si-yu Zhang, Kai-qiang Zhang, Jian Li, Yang Liu, Zhi-hua Yin

**Affiliations:** ^1^Department of Preventive Dentistry, School and Hospital of Stomatology, China Medical University, Liaoning Provincial Key Laboratory of Oral Diseases, Shenyang 110002, China; ^2^Department of Epidemiology, School of Public Health, China Medical University, Shenyang 110122, China

## Abstract

**Background:**

Dental caries is one of the most common chronic diseases observed in elderly patients. The development of preventive strategies for dental caries in elderly individuals is vital.

**Objective:**

The objective of the present study was to construct a generalized regression neural network (GRNN) prediction model for the risk assessment of dental caries among the geriatric residents of Liaoning, China.

**Methods:**

A stratified equal-capacity random sampling method was used to randomly select 1144 elderly (65-74 years) residents (gender ratio 1 : 1) of Liaoning, China. Data for the oral assessment, including caries characteristics, and questionnaire survey from each participant were collected. Multivariate logistic regression analysis was then performed to identify the independent predictors. GRNN was applied to establish a prediction model for dental caries. The accuracy of the unconditional logistic regression and the GRNN early warning model was compared.

**Results:**

A total of 1144 patients fulfilled the requirements and completed the questionnaires. The caries rate was 68.5%, and the main associated factors were toothache history, residence area, smoking, and drinking. We randomly divided the data for the 1144 participants into a training set (915 cases) and a test set (229 cases). The optimal smoothing factor was 0.7, and the area under the receiver operating characteristic curve for the GRNN model was 0.626 (95% confidence interval, 0.544 to 0.708), with a *P* value of 0.002. In terms of consistency, sensitivity, and specificity, the GRNN model was better than the traditional unconditional multivariate logistic regression model.

**Conclusion:**

Geriatric (65-74 years) residents of Liaoning, China, have a high rate of dental caries. Residents with a history of toothache and smoking habits are more susceptible to the disease. The GRNN early warning model is an accurate and meaningful tool for screening, early diagnosis, and treatment planning for geriatric individuals with a high risk of caries.

## 1. Introduction

Dental caries (tooth decay) is one of the most common chronic diseases observed in elderly populations [1–4]. Dental caries is more prevalent in elderly individuals who have a higher risk of losing teeth due to caries than other groups of people [4, 5]. Additionally, poor oral health can have a significant impact on the quality of life for patients and increase the risk of associated systemic chronic diseases [6–8]. Therefore, the development of preventive strategies for dental caries is vital.

China has the largest aging population in the world, accounting for 20% of the global total [9]. However, being a developing country, China has a relative shortage of oral healthcare facilities and medical insurance services are not universal throughout the country [10]. Moreover, elderly individuals have limited access to oral healthcare services, remarkably potentiating the burden of senile dental caries. Four national oral health epidemiological surveys have been conducted periodically (in 1983, 1995, 2005, and 2015) to determine the prevalence and regularity of senile dental caries in China. The caries prevalence and average decayed-missing-filled teeth (DMFT) indices in the Chinese elderly population were reported to be 98.4% and 14.65%, respectively [11, 12]. Additionally, most cases of dental caries (>60%) were concentrated in 20% of participants who were at a high risk of developing the disease [13]. These findings emphasized the urgent need for the prevention and timely management of dental caries among geriatric residents in China. Although Liaoning province is located in the center of northeast China economic zone, the burden of oral diseases is significant among the residents, corresponding to their lower level of awareness about oral health [14]. Identification of the high-risk groups among the geriatric population and management of the disease with targeted therapy provided accordingly can be beneficial to effectively control the development of dental caries.

Current prediction methods for dental caries are based on traditional logistic regression models. However, due to the limitations of the principles of regression, such as that the variables must satisfy the criteria for independence and other assumptions, the logistic regression model cannot address the collinearity problem between variables. Therefore, the application of the logistic regression model for the prediction of caries in the elderly population has its limitations.

An artificial neural network (ANN) is an information processing system that mimics the structure and function of the biological brain [15]. There are four main neural network models currently used in practice: the back-propagation neural network (BPNN), generalized regression neural network (GRNN), fuzzy neural network (FNN), and probabilistic neural network (PNN) [16]. GRNN is a four-layer forward network consisting of an input layer, a hidden layer, a summation layer, and a division layer and has a strong nonlinear mapping ability and accurate prediction capacity. Compared to other models, GRNN has a number of advantages such as approximating ability, classification ability, and learning speed [17]. In addition, GRNN converges on the optimal regression surface with large-sample aggregation that has a reasonable extrapolation and good prediction effects when the sample data is lacking [18]. Therefore, GRNN adapts to the high complexity, nonlinearity, and uncertainty associated with the occurrence and development of dental caries in the elderly population. There are only a few reports on the application of GRNN for disease prediction in the field of stomatology. Zakrzewska et al. used GRNN to study the fluoride distribution in the mandible and teeth [19]. To the best of our knowledge, no published reports describe the utilization of GRNN to establish a prediction model for dental caries in the elderly population. Therefore, the objective of the present study was to construct a GRNN prediction model for the risk assessment of dental caries among the geriatric residents of Liaoning, China. For this purpose, data from a random sample survey of the oral health epidemiology of the elderly population were analyzed.

## 2. Materials and Methods

The present cross-sectional survey study was approved by the institutional review board of the School and Hospital of Stomatology, China Medical University, Liaoning Provincial Key Laboratory of Oral Diseases, Shenyang, China. The data were collected from May to December 2015. Each participant underwent an oral health examination and completed a face-to-face oral questionnaire survey. The data collection and analysis were performed in 2018. The project followed the ethical guidelines provided by the World Medical Association Declaration of Helsinki (2013).

### 2.1. Study Participants

The anticipated sample size was determined based on the sixth edition of “Stomatological Preventive Medicine” [20]. The sample content for the present cross-sectional survey was calculated using the following formula: *N* = *k* × (1 − *P*)/*P*. In this formula, *N* is the sample size, *k* is defined based on the allowable error for the research project, and *P* is the expected prevalence of dental caries. With an allowable error of 10%, a *k* value determined as 400 according to the allowable error size, and an expected prevalence of 67.5% for dental caries (*P*) (the incidence of senile caries in the three northeastern provinces in 2013) [21], the sample size (*N*) required was *N* = 400 × 32.5/67.5 = 193. To ensure an adequate and effective sample size and avoid loss of follow-up or rejection, the recruitment sample size was expanded.

**Figure 1:**
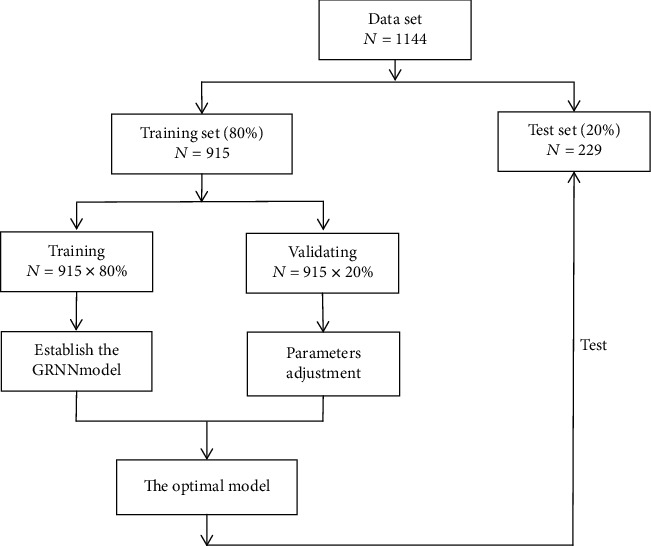
Flowchart representing the development of the generalized regression neural network (GRNN) model.

A stratified equal-capacity random sampling method was used to randomly select 1168 geriatrics (65-74 years; gender ratio 1 : 1) among the urban and rural residents of the Liaoning province, China. The eligibility criteria included age (65-74 years), willingness to cooperate with clinical oral examinations, and residing in the locality of the survey for more than six months. The elderly who were over 75 years old were excluded from the study as they were unable to cooperate with an oral examination, illiterate, or unable to answer the questions in the questionnaire were excluded from the survey. A total of 1144 participants met the required criteria and completed the questionnaires. Informed consent was obtained from each participant (see study diagram in Figure 1).

### 2.2. Assessment of Study Participants

All 1144 study participants received a comprehensive dental examination at the study clinic. The examinations were done by a team of calibrated and trained dentists (Liu, Zhang and Li, Zhang) and included three components:

1. General oral assessment, including dentition status and caries assessment
2. Caries characteristics, including the number of carious lesions and category (crown caries and/or root caries). According to the World Health Organization (WHO), crown caries refers to the formation of an obvious cavitation, enamel destruction, or clear lesions with a softening bottom or wall of the tooth surface, detected during Community Periodontal Index (CPI) probing. Root caries refers to the exposed root showing a soft or leathery lesion, detected with CPI probes [22]. The full list of the related variables is given in Appendix II
3. Through face-to-face interviews, a questionnaire was used to compile the patients' data regarding their age, gender, oral hygiene habits, fluoride toothpaste use, eating pattern, household registration type, oral health behavior, oral medical treatment, education level, and dental health knowledge. See more details in Appendix II

The definitions of predictors were as follows: (a) eating candy frequently: the frequency of eating sweet foods (such as sugary or carbonated drinks, fresh fruits, cookies, cakes, candies, chocolates, sugary milk, and yogurt) was greater than or equal to twice per week. (b) Smoking/drinking habit: elderly participants who had a habit of smoking or drinking daily or weekly were defined as having a habit of smoking or drinking. (c) Use of toothpaste/toothpick/floss was defined as frequent if the usage frequency was greater than twice a week.

### 2.3. Quality Control

The examination standards for dental caries detection in the participants were based on the WHO Basic Methods for Oral Health Survey [22]. One examiner and three questionnaire investigators, who were senior dental specialists, conducted the investigation. All investigators were trained before the start of the formal investigation. The four-member research team conducted a pilot study on a sample population in Liaoning province, China. According to the standard conformance test, the kappa values between the investigators were ~0.85. The on-site formal inspection facilities were the same as those used for the pilot research and included a mobile dental examination chair, illumination lamp, and CPI probes.

### 2.4. Statistical Analysis

Continuous variables were summarized with descriptive statistics in the form of mean and standard deviations (SD). The categorical variables were described as counts and percentages. Univariable Pearson's chi-squared test was used to screen out meaningful factors (*P* < 0.05) that may affect the occurrence of dental caries. Multivariate logistics regression analysis was performed to identify the independent predictors using variables that had a statistical association with the occurrence of dental caries. The odds ratio (OR) and 95% confidence interval (CI) were used to describe the associations.

We randomly divided the data for the 1144 participants into two parts: 80% (915 cases) were included in the training set for the establishment of unconditional logistic regression and the GRNN early warning model, and the remaining 20% (229 cases) were used as the test set for model prediction.

Based on the one-way chi-square data for the training set, statistically significant variables were included in the unconditional logistic regression model using the forward method to establish a multifactor predictive model. The unconditional multivariate logistic regression model was then used to predict whether the subjects in the test set (229 cases) had dental caries.

The statistically significant variables from the chi-square test were used as the input, and the outcome variable (the presence of dental caries) was the output. The GRNN early warning model was established with the neural network toolbox using the MATLAB software (MathWorks, Natick, MA). Afterward, 20% of participants (183 out of 915) were randomly selected from the training set as a special test set to find the optimal smoothing factor for GRNN. The smoothing factor was determined using the method proposed by Sprecht [23]. We increased the unit quantity (0.1) in increments of 0.1 and obtained the respective predicted value for the test set. The optimal smoothing factor was set when the mean square error between the test set predicted value and the sample measured value was the smallest. The receiver operating characteristic (ROC) curve for the model prediction results was plotted. The model prediction probability value corresponding to the maximum value of Youden's index (sensitivity + specificity-1) during training was set as the best cut-off value for the present study. The comparison between the unconditional logistic regression and GRNN models was performed according to the statistical analysis of consistency, sensitivity, specificity, and area under the ROC curve. Finally, the optimal cut-off value for the predictive probability of the logistic regression model in the present study was determined to be 0.606, with a corresponding Youden's index of 0.370. The optimal cut-off value for the GRNN model's predictive probability was 0.680, with a corresponding Youden's index of 0.638. *P* values less than 0.05 were considered statistically significant for all tests. Statistical analyses of all the data except the GRNN model were performed using SPSS 22.0 (BMI Corporation, Chicago, IL, USA).

## 3. Results

### 3.1. Characteristics of the Study Participants

The participants included in the final analysis consisted of 1,144 individuals aged 68.3 ± 3.1 years (mean ± SD) with an equal gender distribution (male and female) and residence area (urban and rural) (see Table 1). A total of 784 (68.5%) participants had dental caries, while 360 (31.5%) did not. The details of the demographic characteristics, including the lifestyle, beliefs, and behaviors of the included participants, are shown in Table 1.

### 3.2. Correlation Test for Each Target Factor

The correlation test results for each target factor (Appendix II) for the occurrence of dental caries in the participants are shown in Table 2. The univariable analysis showed that the targeted factors (e.g., residency, use of a removable upper jaw dental prosthesis, use of a removable lower jaw dental prosthesis, number of natural teeth, having toothache in previous years, smoking, drinking alcohol, domestic access to tap water, use of toothpicks, healthcare service needs, believing oral health has an impact on eating, self-oral health assessment, and self-oral hygiene assessment) had a statistical association with the occurrence of dental caries (*P* < 0.05). Other factors including gender, age, dental insurance, eating candy often, visiting the dental clinic for caries without pain, drinking carbonated beverages, use of dental floss and fluoride toothpaste, and visiting the dental clinic in the past year showed no statistical association (Table 2).

### 3.3. Multivariate Logistic Regression Results

The independent predictors for dental caries were identified based on multivariate logistics regression analysis (Table 3). In elderly individuals, a history of toothache in previous years (yes vs. no, OR = 1.550, 95% CI: 1.164-2.063), use of a removable upper jaw dental prosthesis (yes vs. no, OR = 4.320 with 95% CI: 2.647 to 7.051), use of a removable lower jaw dental prosthesis (yes vs. no, OR = 4.420 with 95% CI from 2.477 to 7.885), living in a rural area (urban area vs. rural area, OR = 0.676 with 95% CI from 0.503 to 0.908), smoking (yes vs. no, OR = 1.469, 95% CI: 1.084-1.992), and drinking alcohol (yes vs. no, OR = 1.591 with 95% CI from 1.130 to 2.240) were predictors for dental caries. On the other hand, good self-oral hygiene evaluation (good vs. not good, OR = 0.606, 95% CI: 0.423-0.868) was a protective factor for participants against dental caries. We evaluated the potential collinearity between the variables, and the variance inflation factor (VIF) corresponding to these variables was less than 2, suggesting that there was no multicollinearity problem in the model.

### 3.4. Dental Caries Prediction Models

We randomly divided the data for the 1144 participants into two parts: 80% (915 cases) were included in the training sets for the establishment of unconditional logistic regression and the GRNN early warning model, while the remaining 20% (229 cases) were used as test sets for model prediction (see Figure 1). The data collected from the training set (80%, 915 cases) were tested with a one-way chi-square test, and 15 variables were found to be related to the occurrence of dental caries in the elderly population (Appendix I). These variables included the residence area (*P* = 0.002), upper and lower removable dental prosthesis (*P* < 0.001), number of true teeth and having toothache in previous years (*P* < 0.001), smoking (*P* < 0.001), number of cigarettes per day (*P* = 0.003), drinking alcohol (*P* = 0.006), eating candy frequently (*P* = 0.0034), drinking carbonated beverages frequently (*P* = 0.023), domestic water access (*P* = 0.005), use of toothpicks (*P* = 0.004), believing that oral health has an impact on eating (*P* = 0.001), self-oral health assessment (*P* = 0.007), and self-oral hygiene assessment (*P* = 0.001).

**Figure 2:**
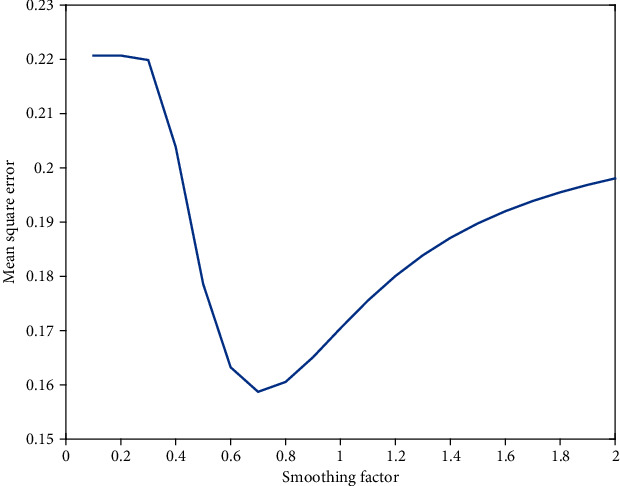
Determination of mean square error and optimal smoothing factor.

According to the unconditional multivariate logistic regression, seven variables (residence area, number of cigarettes/day, using a removable upper/lower jaw dental prosthesis, toothache in previous years, and drinking carbonated beverages and alcohol) were entered into the model (Table 4). The fitting and prediction results for the model using the subjects in the training and test sets (229 cases) are compared in Table 5. The results showed an optimal smoothing factor of 0.7 (Figure 2). Seven statistically significant variables from the chi-square test were used as the input, and the outcome variable (the presence of caries in elderly participants) was used as the output. The fitting and prediction results for the GRNN model using the subjects in the training and test sets are shown in Table 6.

### 3.5. Comparison between the Unconditional Logistic Regression and GRNN Models

A comparison of the accuracy of the unconditional logistic regression and GRNN models showed that the classification consistency, sensitivity, and specificity of the GRNN model were higher than those of the unconditional logistic regression model (Table 7).

**Figure 3:**
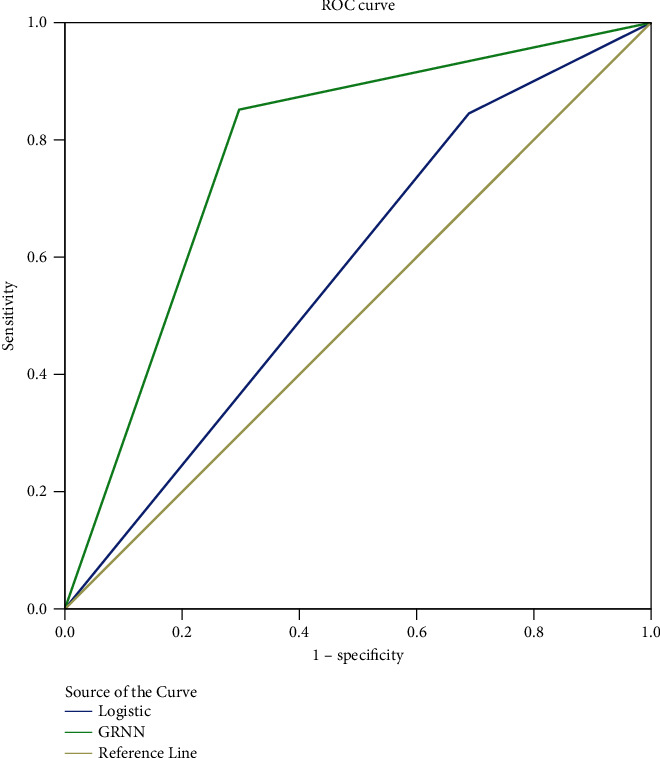
ROC curves for the logistic regression and GRNN models in the data set with the best cut-off values.

The optimal cut-off value for the predictive probability of the logistic regression model in the present study was found to be 0.606, with a corresponding Youden's index of 0.370. The optimal cut-off value for the GRNN model's predictive probability was 0.680, with a corresponding Youden's index of 0.638. The ROC curves for the logistic regression and GRNN models generated using the data set with the best cut-off values are shown in Figure 3. The areas under the ROC curves for the logistic regression and GRNN models were 0.578 and 0.777, respectively, with corresponding *P* values of 0.056 and < 0.001 compared to the baseline. The *P* value for the comparison of the area under the ROC curve for the two models was < 0.001. The GRNN model was better than the unconditional logistic regression model in terms of accuracy.

## 4. Discussion

In the present study, a GRNN predicting model was used for dental caries risk assessment among geriatric residents. For this purpose, we investigated 1144 old (65-74 years) residents of Liaoning province, China, and performed a comprehensive statistical analysis comparing the accuracy of unconditional logistic regression and GRNN models. The caries rate in the geriatric population was 68.5%, which is similar to the survey results from the third oral health epidemiological investigation in the Liaoning province performed 11 years ago [12]. These findings suggested that oral healthcare for the old residents of this province had not received enough attention and suffered from a lack of corresponding oral healthcare publicity and education in recent decades. There are a number of factors associated with compromised oral healthcare in the region. For example, although the living standards have improved in recent years, the eating habits of elderly individuals have not changed. Our results showed that the use of dental floss (0.3%) and fluoride toothpaste (29.5%) and the proportion of participants who sought medical treatment (27.0%) for oral diseases were remarkably low. Lack of patient education and understanding of oral health management also contributed to poor oral healthcare. The financial burden is also an essential factor in the development of caries as most elderly residents in the Liaoning province have to pay for oral healthcare services [14, 20].

The present study showed that the main risk factors associated with elderly dental caries were toothache history, residence type, and smoking and drinking status. These factors were slightly different from those identified in the 3^rd^ oral health epidemiological investigation, which indicated that using fluoride toothpaste, consuming sugary foods, frequency of teeth brushing, and the area of residence are factors related to dental caries that may affect the Chinese geriatric population (65-74 years) [12]. In contrast, we did not find a statistical significance in using fluoride toothpaste and eating sugar-containing foods with elderly dental caries. This may be due to the low utilization of fluoride toothpaste and sugar-containing foods in the studied population; only 29.5% (338/1144) used fluoride-containing toothpaste, and only a few participants were able to eat sugary desserts or beverages on a daily basis.

Smoking and drinking are common risk factors for several chronic oral conditions, including gingivitis, periodontitis, and gingival recession among elderly dental patients [24]. Plaque adherence to the cementoenamel junction and root surface can induce changes in the cementum, leading to demineralization, decay, and root caries [25]. The synergistic effects of drinking and smoking are also likely to increase the risk of oral diseases in elderly individuals, further enhancing the occurrence and development of dental caries [20].

Using removable dental prosthesis in the upper jaw (OR (95% CI), 4.320 (2.647-7.051)) and lower jaw (OR (95% CI), 4.420 (2.477-7.885)) had a significant influence on the occurrence of dental caries in the elderly participants. These findings are in agreement with previous studies [1, 26–28]. The use of a removable prosthesis may facilitate the growth of cariogenic bacteria by changing the oral environment, increasing the risk of developing dental caries [28, 29]. Therefore, for patients who wear removable dental prosthesis, dental healthcare professionals should be alert for the possibility of dental caries and take measures to prolong the survival of teeth.

Another important risk factor associated with the occurrence of dental caries in the elderly population is self-oral hygiene assessment. A better capability to assess self-oral hygiene resulted in the reduction of dental caries (*P* = 0.005). Patients with good oral hygiene are confident in their self-oral hygiene assessment [30].

The present study found that participants with a history of toothache were more susceptible to dental caries, which is consistent with the findings reported by Liu et al. [31]. Elderly patients suffering from toothache usually endure pain rather than visiting the dentist to avoid the inconvenience or due to economic factors. Over an extended period, such an attitude may lead to the development of dental caries and associated dental pain [32]. Living in rural areas was a protective factor for elderly caries, which is consistent with previous studies [33, 34]. With the rapid development of urban economies, various factors such as the living standards, eating habits, and sugar consumption of urban elderly residents have become remarkably different from those of the residents of rural areas. For instance, the type of food intake is more diverse than that in rural areas, and most consumed foods are finely processed products. Consequently, the daily dietary sugar intake of urban elderly residents is higher than that of rural residents. Additionally, rural residents in this province usually like to eat dietary fiber-rich foods. Nutritional fiber-rich foods have an oral self-cleaning effect, thus protecting against the development of caries [35, 36].

GRNN was applied in the present study to establish a prediction model for dental caries in the elderly population. Compared to the traditional BPNN, the training process for GRNN does not need to be iterative, and the training parameters for the network only have an optimal smoothing factor [37]. After the optimal smoothing factor is determined, the predictive ability is stable. In the present study, 20% of the training set was randomly selected as the test set to find the optimal smoothing factor for the generalized regression neural network, and good extrapolation of the prediction results was ensured. In terms of variable selection, we first used the single-factor chi-square test to reduce the dimensions of modeling variables to ensure the simplicity of the model and prevent the existence of too much redundant information from reducing the prediction effects.

In the present study, we found that the consistency, sensitivity, and specificity of the GRNN model were better than those of the traditional unconditional multivariate logistic regression model in the training and test sets. The area under the ROC curve for the GRNN model was also larger than that of the unconditional multivariate logistic regression model, with better prediction results. However, the shortcomings of the GRNN model were also apparent. According to the OR value obtained in the unconditional multivariate logistic regression model, we can explain the results professionally and roughly judge the degree of influence from the variables on the results, which is not possible using a GRNN model. GRNN acts like a “black box,” which makes it difficult for us to interpret the results from a professional point of view and to judge the contribution of variables. GRNN has no way to calculate the *P* and OR values for each included variable, so there is no way to determine the contribution of GRNN or to explain the risk of disease. Further, GRNN can only provide the results of disease prediction. GRNN can predict diseases, but it is impossible to determine which of the variables included in the prediction model play a key role. Therefore, compared with traditional methods, the GRNN model is more accurate for prediction, but is less explanatory. This is common for all in-depth learning models. Another limitation of the current cross-sectional study is that the factors used to create the GRNN model may not be accurate predictors as the study is not a cohort study. Additionally, reading limitations for patients may have introduced bias while they completed the questionnaire. Future research shall focus on patient follow-up and other important factors (e.g., systematic diseases) to assess the validity of the tool.

## 5. Conclusion

The geriatric (65-74 years) residents of Liaoning, China, presented a high rate of dental caries. Residents with a history of toothache and smoking habits were more susceptible to dental caries. The GRNN model had better consistency, sensitivity, and specificity than traditional unconditional multifactor logistic regression models in both the training and test sets. Therefore, the GRNN early warning model is an accurate and meaningful tool for screening, early diagnosis, and treatment planning for elderly individuals with a high risk of dental caries. Simultaneously, further measures should be taken to promote oral healthcare education for the elderly population and establish a sound primary oral healthcare system.

## Figures and Tables

**Table 1:** The demographic and lifestyle characteristics of the study participants (*n* = 1144).

Characteristic	Value
Demographic characteristics	
Age (mean ± SD), years	68.29 ± 3.122
Female, *n* (%)	576 (50.3)
The number of people with caries, *n* (%)	784 (68.5)
Residence area	
Urban, *n* (%)	573 (50.1)
Rural, *n* (%)	571 (49.9)
Use of a removable upper jaw dental prosthesis	
Yes, *n* (%)	156 (13.6)
No, *n* (%)	988 (86.4)
Use of a removable lower jaw dental prosthesis	
Yes, *n* (%)	123 (10.8)
No, *n* (%)	1021 (89.2)
Number of true teeth	
>*n*?	730 (63.8)
≤*n*	414 (36.2)
Dental insurance	
Yes, *n* (%)	144 (12.6)
No, *n* (%)	1000 (87.4)
Having toothache in previous years	
Yes, *n* (%)	535 (46.8)
No, *n* (%)	609 (53.2)
Lifestyle	
Smoking	
Yes, *n* (%)	737 (64.4)
No, *n* (%)	407 (35.6)
Drinking alcohol	
Yes, *n* (%)	907 (79.3)
No, *n* (%)	237 (20.7)
Eating candy frequently	
Yes, *n* (%)	72 (6.3)
No, *n* (%)	1072 (93.7)
Drinking carbonated beverages frequently	
Yes, *n* (%)	32 (2.8)
No, *n* (%)	1112 (97.2)
Domestic water access	
Tap water, *n* (%)	732 (64.0)
Not tap water, *n* (%)	412 (36.0)
Use of toothpick	
Yes, *n* (%)	720 (62.9)
No, *n* (%)	424 (37.1)
Use of dental floss	
Yes, *n* (%)	4 (0.3)
No, *n* (%)	1140 (99.7)
Use of fluoride toothpaste	
Yes, *n* (%)	338 (29.5)
No, *n* (%)	806 (70.5)
Beliefs and behaviors	
Healthcare service utilization	
Yes, *n* (%)	749 (65.5)
No, *n* (%)	395 (34.5)
Believing oral health has an impact on eating	
Yes, *n* (%)	641 (56.0)
No, *n* (%)	503 (44.0)
Visiting dental clinic for caries without pain	
Yes, *n* (%)	296 (25.9)
No, *n* (%)	848 (74.1)
Visiting dental clinic in the past year	
Yes, *n* (%)	193 (16.9)
No, *n* (%)	951 (83.1)
Visiting dental clinic for toothache	
Yes, *n* (%)	309 (27.0)
No, *n* (%)	835 (73.0)
Self-oral health assessment	
Not good, *n* (%)	447 (39.1)
Good, *n* (%)	697 (60.9)
Self-oral hygiene assessment	
Not good, *n* (%)	258 (22.6)
Good, *n* (%)	886 (77.4)

**Table 2:** The prevalence of dental caries and the studied variables.

Characteristic	The number of participants with caries	The number of participants without caries	*^^^ P* value
Demographic characteristics			
Age			
<70	494	248	
≥70	290	112	*P* = 0.303
Gender			
Female, *n* (%)	399	177	
Male, *n* (%)	385	183	*P* = 0.588
Residency			
Urban, *n* (%)	419	154	
Rural, *n* (%)	365	206	*P* = 0.001
Use of a removable upper jaw dental prosthesis			
Yes, *n* (%)	37	119	
No, *n* (%)	747	241	*P* < 0.001
Use of a removable lower jaw dental prosthesis			
Yes, *n* (%)	23	100	
No, *n* (%)	761	260	*P* < 0.001
Dental insurance			
Yes, *n* (%)	97	47	
No, *n* (%)	688	312	*P* = 0.745
Number of true teeth			
>20	537	193	
≤20	247	167	*P* < 0.001
Having toothache in previous years			
Yes, *n* (%)	417	118	
No, *n* (%)	367	242	*P* < 0.001
Lifestyle			
Smoking			
Yes, *n* (%)	538	199	
No, *n* (%)	246	161	*P* < 0.001
Drinking alcohol			
Yes, *n* (%)	268	639	
No, *n* (%)	92	145	*P* = 0.006
Eating candy frequently			
Yes, *n* (%)	42	30	
No, *n* (%)	742	330	*P* = 0.054
Drinking carbonated beverages frequently			
Yes, *n* (%)	18	14	
No, *n* (%)	766	346	*P* = 0.129
Domestic water access			
Tap water, *n* (%)	525	207	
No tap water, *n* (%)	259	153	*P* = 0.002
Use of toothpick			
Yes, *n* (%)	468	252	
No, *n* (%)	316	108	*P* = 0.001
Use of dental floss			
Yes, *n* (%)	4	0	
No, *n* (%)	780	360	*P* = 0.175
Use of fluoride toothpaste			
Yes, *n* (%)	242	96	
No, *n* (%)	545	261	*P* = 0.152
Attitudes and behaviors			
Healthcare service needs			
Yes, *n* (%)	531	218	
No, *n* (%)	253	142	*P* = 0.018
Believing oral health has an impact on eating			
Yes, *n* (%)	474	167	
No, *n* (%)	310	193	*P* < 0.001
Visiting dental clinic for caries without pain			
Yes, *n* (%)	206	90	
No, *n* (%)	578	270	*P* = 0.647
Visiting dental clinic in the past year			
Yes, *n* (%)	132	61	
No, *n* (%)	652	299	*P* = 0.964
Self-oral health assessment			
Not good, *n* (%)	289	158	*P* = 0.024
Good, *n* (%)	495	202	
Self-oral hygiene assessment			
Not good, *n* (%)	199	59	*P* = 0.001
Good, *n* (%)	585	301	

^^^Pearson's chi-square test.

**Table 3:** The independent predictors according to the multivariate logistic analysis.

Characteristic	OR (95% confidence interval)	*P* value
Resident area		
Urban vs. rural	0.676 (0.503–0.908)	*P* = 0.009
Use of a removable upper jaw dental prosthesis		
Yes vs. no	4.320 (2.647–7.051)	*P* < 0.001
Use of a removable lower jaw dental prosthesis		
Yes vs. no	4.420 (2.477–7.885)	*P* < 0.001
Having toothache in previous years		
Yes vs. no	1.550 (1.164–2.063)	*P* = 0.003
Smoking		
Yes vs. no	1.469 (1.084–1.992)	*P* = 0.013
Drinking alcohol		
Yes vs. no	1.591 (1.130–2.240)	*P* = 0.008
Self-oral hygiene assessment		
Good vs. not good	0.606 (0.423-0.868)	*P* = 0.005

Abbreviation: OR for odds ratio.

**Table 4:** The independent predictors according to the unconditional multivariate logistic regression.

Characteristic	OR (95% confidence interval)	*P* value
Resident area		
Urban vs. rural	0.656 (0.475-0.906)	*P* = 0.011
Number of cigarettes per day	0.652 (0.443-0.957)	*P* = 0.029
Use of a removable upper jaw dental prosthesis		
Yes vs. no	5.655 (3.242-9.864)	*P* < 0.001
Use of a removable lower jaw dental prosthesis		
Yes vs. no	5.808 (3.025-11.152)	*P* < 0.001
Having toothache in previous years		
Yes vs. no	1.628 (1.174-2.256)	*P* = 0.003
Drinking carbonated beverages frequently		
Yes vs. no	0.270 (0.113-0.644)	*P* = 0.003
Drinking alcohol		
Yes vs. no	0.523 (0.359-0.760)	*P* = 0.001

Abbreviation: OR for odds ratio.

**Table 5:** The fitting and prediction results of the logistic regression model for the subjects in the training and test sets.

Predictive results	Training set		Test set	
	Number of true caries	Number of true no caries	Number of true caries	Number of true no caries
+	565	151	131	51
-	64	135	24	23
Total	629	286	155	74

**Table 6:** The fitting and prediction results of the generalized regression neural network (GRNN) model for the subjects in the training and test sets.

Predictive results	Training set		Test set	
	Number of true caries	Number of true no caries	Number of true caries	Number of true no caries
+	575	79	132	22
-	54	207	23	52
Total	629	286	155	74

**Table 7:** Comparison of the accuracy between the unconditional logistic regression model and the generalized regression neural network (GRNN) model.

Models	Training set			Test set		
	Consistency	Sensitivity	Specificity	Consistency	Sensitivity	Specificity
Logistic	76.50%	89.83%	47.20%	67.25%	84.52%	31.08%
GRNN	85.46%	91.41%	72.38%	77.29%	85.16%	70.27%
*χ* ^2^	21.888	0.988	57.284	11.758	0.000	25.290
*P* value	0.001	0.320	0.001	0.001	1.000	0.001

## Data Availability

The datasets used and/or analyzed during the current study are available from the corresponding author on reasonable request.

## Abbreviations

ANN: Artificial neural network
GRNN: Generalized regression neural network
BPNN: Back-propagation neural network
FNN: Fuzzy neural network
PNN: Probabilistic neural network.

## Supplementary Materials

Supplementary 1 Appendix I: variables related to the occurrence of dental caries in elderly individuals according to the training set data.

Supplementary 2 Appendix II: the full list of variables assessed in the present study.
