# Supplementary material for: Dental Caries Prediction Based on a Survey of the Oral Health Epidemiology among the Geriatric Residents of Liaoning, China
Source: Biomed Res Int. 2020 Dec 7;2020:5348730. doi: 10.1155/2020/5348730 (PMC7739046; doi:10.1155/2020/5348730)
Supplement: Supplementary 1 — Appendix I: variables related to the occurrence of dental caries in elderly individuals according to the training set data. [file 5348730.f1.docx]

Appendix I. Variables related to the occurrence of dental caries in elderly individuals according to the training set data.

| Variables | *χ*^2^ | *P value* |
| --- | --- | --- |
| Residence area | 9.824 | 0.002 |
| Number of natural teeth | 16.731 | <0.001 |
| Using a removable upper jaw dental prosthesis | 147.047 | <0.001 |
| Using a removable lower jaw dental prosthesis | 131.731 | <0.001 |
| Eating candy frequently | 4.493 | 0.034 |
| Drinking carbonated beverages frequently | 5.147 | 0.023 |
| Smoking | 12.150 | <0.001 |
| Number of cigarettes daily | 13.761 | 0.003 |
| Drinking | 7.594 | 0.006 |
| Using toothpick | 8.183 | 0.004 |
| Having toothache in previous years | 35.395 | <0.001 |
| Belief that food having influence on oral health | 10.830 | 0.001 |
| Self-health assessment | 7.248 | 0.007 |
| Self-oral health assessment | 8.517 | 0.004 |
| Domestic water access | 7.763 | 0.005 |
