# Supplementary material for: Dental Caries Prediction Based on a Survey of the Oral Health Epidemiology among the Geriatric Residents of Liaoning, China
Source: Biomed Res Int. 2020 Dec 7;2020:5348730. doi: 10.1155/2020/5348730 (PMC7739046; doi:10.1155/2020/5348730)
Supplement: Supplementary 2 — Appendix II: the full list of variables assessed in the present study. [file 5348730.f2.docx]

**Appendix II.** The full list of variables assessed in the present study.

ID number

Name

Sex

Age

Date of investigation

Number of investigators

Number of remaining true teeth

Removable lower jaw dental prosthesis (yes/no)

Removable upper jaw dental prosthesis (yes/no)

Frequency of having carbonated foods or drinks (e.g., fresh fruit, dessert, chocolate candy, sugar beverage, juice)

Smoking status (yes/no)

Smoking (yes/no)

Drinking alcohol (yes/no)

Drinking tea (yes/no)

Frequency of flossing, using toothpicks, and brushing teeth

Types of toothpaste (fluoride toothpaste or not)

History of toothache (yes/no)

Actions taken to deal with the last toothache (went to the dental clinic or not)

Dental expenditure (dental insurance or not)

Duration since the last visit to the dentist

Duration since the last visit to the clinic for a tooth ache

Reasons for not visiting the clinic for a tooth ache

Number of dental clinic visits over the past year

Reasons for the last visit to the dental clinic

Choice of dental clinics (public clinics or private clinics)

Last denture treatment

Cost of the last dental visit

Cost of dental drugs from the last dental visit

Perceived oral health effects on eating, pronunciation, appearance, self-esteem, and social interactions (yes/no)

Assessment of general health, dental health, gum health, and oral hygiene

Access to water in the home (domestic water or not)

Number of people living with the family

Belief that food having influence on oral health

Annual income of the family
